# The multidimensionality of female mandrill sociality—A dynamic multiplex network approach

**DOI:** 10.1371/journal.pone.0230942

**Published:** 2020-04-13

**Authors:** André S. Pereira, Inês D. Rebelo, Catarina Casanova, Phyllis C. Lee, Vasilis Louca

**Affiliations:** 1 School of Biological Sciences, University of Aberdeen, Aberdeen, United Kingdom; 2 Research Centre for Anthropology and Health, Department of Life Sciences, University of Coimbra, Coimbra, Portugal; 3 CAPP, Centro de Administracão e Políticas Públicas, School of Social and Political Sciences, University of Lisbon, Lisbon, Portugal; 4 Psychology Division, Faculty of Natural Sciences, University of Stirling, Stirling, United Kingdom; University of Tasmania, AUSTRALIA

## Abstract

The structure and dynamics of primate social groups are shaped by the social relationships of its members. These relationships are based on different types of interactions and vary in relation to the identity of the interactants and over time. Social network analysis tools represent a powerful and comprehensive method to characterise social interactions and recent methodological advances now allow the study of the multidimensionality of sociality via multilayer networks that incorporate multiple types of interactions. Here, we use a multidimensional network approach to investigate the multidimensionality of sociality of females in a captive group of mandrills. We constructed two multiplex networks based on agonistic, proximity and grooming interactions of 6–7 mature females to analyse the multidimensionality of relationships within two independent observation periods; and three multiplex networks (one for each interaction type) to examine how relationships changed between periods. Within each period, different individuals were the most central in each layer and at the multiplex level, and different layers (i.e., interaction types) contributed non-redundant information to the multilayer structure. Across periods, relationships based on the same interaction type also contained non-redundant information. These results indicate that female mandrills engage in multidimensional and dynamic relationships, suggesting that in order to represent the full complexity of relationships, networks need to be constructed from more than a single type of interaction and across time. Our results provide evidence for the potential value of the multilayer network approach to characterise the multidimensionality of primate sociality.

## Introduction

Most primates form socially complex groups [1], in which individuals interact using a variety of behaviours. Sociality is theoretically conceived of as the result of a trade-off between the advantages and disadvantages of group life, such as decreased predation risk and increased competition for resources [2–3], although the measurement of costs and benefits to individuals remains problematic even after 40 years [4]. The quantity and quality of an individual’s social relationships, as well as its integration into its group’s social network, can result in net fitness benefits (e.g., baboons (*Papio* spp): [5–7]; mandrills (*Mandrillus sphinx*): [8]; rhesus macaques (*Macaca mulatta*): [9]). Relationships are an emergent property of interactions between individuals and shape the structure and dynamics of animal societies [10–11]. Since interactions vary temporally and spatially and take place across different social contexts (e.g., affiliative, sexual, parenting and agonistic), relationships are often multidimensional [10].

Due to their potentially adaptive value and to their central role in defining the dynamics and structure of animal societies, primatologists have focused on characterising and understanding how social relationships are structured and regulated. In particular, the social relationships of female Old World monkeys have been among the focus of interest of primatologists [1]. Female Old World monkeys typically remain in their natal group whereas males migrate between groups; hence, females have increased opportunities to develop strong social bonds with each other [1]. There is evidence that female Old World monkeys are capable of making strategic management decisions about their relationships as they maintain stable long-term relationships and more variable ones with different partners [12]. Furthermore, there is evidence that they can adjust their social relationships over short periods of time [13] and in response to seasonality [14–15] or demographic variation [16–17].

Nevertheless, the multidimensionality of primate social relationships and the factors that affect their dynamics remain poorly understood for some species, such as mandrills. Given the importance of social relationships to group structure and to the fitness of individuals, it is thus relevant and timely to expand our understanding of the social dynamics of female Old World monkeys to those species that remain poorly explored. Here, we aim to investigate mandrill interactions and their emergent relationship properties through a multidimensional approach that includes different types of interactions, as well as how these develop and vary through time in the light of demographic variation [18].

Over the last decade, social network analysis (SNA) has emerged as the most comprehensive tool to investigate the dynamics of social relationships at the individual and group level in primate and other animal societies [19–20]. SNA offers unique possibilities, including the characterisation of patterns of interaction across social context and time, the quantification of structural properties of social topology, and the precise identification of which individuals and relationships are central to the social dynamics of a group while being sensitive to the direct and indirect connections between individuals [20]. These unique characteristics make SNA the most comprehensive and powerful method available to investigate social relationships. Whereas the most common social network approaches tend to focus on a single type of interaction (e.g., grooming: [21]; spatial proximity: [22]) or to analyse different interaction types via aggregated networks or individual networks separately [e.g., 23–24], recent developments now allow for the analysis and representation of multiple interconnected networks into a single mathematical object [25–26]. This methodological advance facilitates the study of the multidimensional nature of primate social relationships.

SNA typically considers individuals (nodes) and their interactions (the edges that connect the nodes in the network) [19], and multilayer networks result from the connection of different individual networks (monolayers) through interlayer edges [27–28]. Among the different types of multilayer networks, multiplex networks are those in which interlayer edges connect the same node across different layers [26, 29]. In multiplex networks, each layer can represent a different kind of interaction (e.g., grooming, aggression and proximity) or time period and each node (i.e., each individual) is connected to itself across the different layers where it is found [26, 29].

Multiplex network analysis expands on standard SNA when it comes to investigating the multidimensionality of sociality. Standard SNA approaches measure the social importance of individuals by calculating their centrality to the network of a single interaction type [11, 30–32], or, when analysing more than one type of interaction, ignore the potential interdependence between different types of interactions and lose information on how each type of interaction contributes to the result [29, 33]. Multiplex analysis allows for the calculation of versatility, the multiplex network equivalent to centrality, which measures the impact of an individual across various interaction types [25–26]. This measure better identifies individuals that are actually central in their group but whose centrality may be overlooked in monolayer networks, while retaining information about all individual layers, thus constituting a more appropriate measure of the importance of an individual to its group than centrality [25–26, 33]. Furthermore, by combining individual networks based on different measures, multilayer approaches buffer the consequences of missing interactions that actually take place [25], which have the potential to modify overall network properties [25, 34], allowing for a more accurate representation of relationships among individuals.

The usefulness of multiplex networks in studying primate social structure has been demonstrated by Smith-Aguilar et al. [25]. Using a six-layer multiplex network based on grooming, embraces, aggression, proximity, contact and association, Smith-Aguilar et al. [25] characterised the social structure of a group of wild Geoffroy’s spider monkeys (*Ateles geoffroyi*). The multilayer network approach improved on the standard network approach by retaining both the patterns and the interdependencies of the different kinds of interactions, providing a more accurate representation of social relationships and of the social importance of individuals [25]. Smith-Aguilar et al. [25] showed that even the reduction of the number of layers of their network by one resulted in loss of information, justifying the use of a multidimensional approach to investigate questions about the structure of primate networks and how it emerges from individual interactions.

Mandrills are large-bodied, female-bonded highly gregarious Old World monkeys. In the wild, mandrills may form non-nested societies consisting of a core of females and their offspring. Mandrills form groups of variable size, with a mean group size of 620 individuals for wild hordes in Lopé Reserve [35], that travel the thick equatorial forest of Central Africa in cohesive aggregations [36]. The presence of adult males appears to vary among groups, but they usually leave the group at adolescence and only loosely associate with females after that except when breeding [35–38]. Given the large size of wild mandrill groups, it is likely that females have to keep track of multiple social relationships and that these relationships are dynamic [e.g., 36, 39].

Since individual identification in wild populations of mandrills is challenging, most studies on social networks in this species have been on captive animals [22, 40–41]. Using association networks, females were found to be more central than males and to sustain group cohesion [22]; spatial proximity of individuals to a feeding area was explained by association subgroups as well as by dominance rank, with higher-ranking individuals being more often observed near the feeding area [40]; and individuals of the same sex and relatedness were found to be more likely to associate with each other [22, 40]. Using grooming networks, female centrality was found to vary over time in accordance with the predictions of the biological market theory [41].

Here, we investigate the multidimensionality of relationships among mature females in a small captive matriline of seven maternally related female mandrills, within and across two separate observation periods, using a multiplex network approach based on agonistic, proximity and grooming interactions. The study group went through demographic changes between periods, namely the sexual maturation of a female and the transition of an infant to weaning. These changes influenced the grooming network of the adult females [41] and have the potential to affect their relationships through other types of interactions [17].

We hypothesise that, as observed in other social primates, the social relationships of female mandrills are multidimensional (olive baboons (*Papio anubis*): [23]; wild Geoffroy’s spider monkeys: [25]) and dynamic over time (rhesus macaques: [15]; vervet monkeys (*Chlorocebus pygerythrus*): [17]). We predict that, in a network structure, **the multidimensional dynamics of female mandrill sociality are more accurately represented by (1) considering all three types of interactions separately within the same observation period** [25], and **(2) considering each type of interaction separately for each observation period** (i.e., over time) [17]. We present descriptive data on edge overlap across layers, and on how individual levels of centrality and versatility differ across layers and at the multiplex levels in order to highlight how the structure of layers emerges from individual variation.

## Materials and methods

### Study group and observation periods

We collected data on a colony of 12–13 mandrills housed in Badoca Safari Park, Portugal (38°02’26.5”N 8°44’35.8”W). The colony of mandrills was housed in an enclosure that consisted of an outdoor enclosure of approximately 1674 m^2^ and an indoor enclosure of approximately 75m^2^. In the morning, after the zookeepers distributed fruit, seeds and vegetables across the outdoor enclosure, the mandrills were let out. The group returned inside in the afternoon, after vegetables, fruit and monkey chow were made available in the indoor enclosure. Water was available *ad libitum*. We only collected data when the group was in the outdoor enclosure.

All individuals were born in captivity and were fully habituated to the presence of humans. Authorisation to study the group was granted by the Animal Department of the park. We collected only non-invasive data through observations carried out in the public area of the park, and all procedures were performed in accordance with the ASAB guidelines for the observation of animals [42] and the European law on humane care and use of laboratory animals.

We collected data in two separate short periods in order to assess the dynamics and stability of female relationships while avoiding confounding variation of social network change within each period [43]. In *period one* we collected data from the 26^th^ of May to the 1^st^ of July of 2016 and in *period two* we collected data between the 26^th^ of January and the 8^th^ of February of 2018.

Our focal animals were the mature females of the group: Mirinda, Nefertari, Camila, Limbe, Lisala, Lolaya and Tania, who were all biologically related, forming a single matriline. The age of all females was known. In both observation periods, all focal females were under contraception via a gonadotrophin-releasing hormone agonist implant. This contraceptive implant is believed to have no direct effect on the social behaviour of female Old World monkeys [44]. Lolaya gave birth more than fifteen weeks after the first period of data collection, indicating that she was pregnant during *period one* despite being on contraception. Lolaya’s infant foraged and travelled independently of his mother in *period two*. Nevertheless, the implant minimised the influence of cycling on the social interactions of the females. Tania was sexually immature in *period one* so we only considered her in *period two*. Before *period one*, Nefertari had given birth to a male infant, who was still dependent on her for travel and food during *period one*. Ten days after data collection ceased in *period two*, Nefertari died of a cardiopulmonary arrest. Her autopsy found no indication of infection. Given that the study group was under a controlled physical and nutritional environment in both observation periods, we expected no effect of seasonality in the social behaviour of the focal females.

### Data collection

We collected data using 15-minute-long individual focal samples [45–46], continuously recording the duration and timing of all behaviours by the focal individual. All interactions between the focal individual and other group members, their duration and the identity of the actor(s) and recipient(s) of interactions were noted. Order of observation of focal individuals was randomised for each day of data collection. When necessary, we used 10 x 20 binoculars. In *period one*, we collected 96 hours of focal observations equally distributed across the six sexually mature females (16 hours per female). In *period two*, we collected 73.5 hours of focal observations equally distributed across the seven sexually mature females (10.5 hours per female).

### Interaction types

We constructed the multiplex networks based on three different interactions: grooming (affiliative measure, measured in total time individuals in dyads spent grooming each other across all grooming events), proximity when feeding (associative/ tolerance measure, measured in total time individuals in dyads spent at arm’s reach of each other across all feeding and foraging events, based on [47]) and agonism (measured by frequency of aggression, supplant and avoidance episodes between individuals in dyads) (S1 Table). Grooming and proximity are considered meaningful measures of the quality of relationships in primates [48–50], whereas agonism defines competitive relationships [51] potentially affecting the fitness of an individual [52–54].

For each type of interaction, we calculated a dyadic index that allowed us to determine the relative strength of each dyadic interaction while avoiding having each layer influencing the multiplex network differently due to scale effects (e.g., grooming and proximity were common while agonistic interactions were rare) [25]. For directed interactions (i.e., grooming and agonism), we summed all occurrences between members of a dyad, regardless of who was the actor and the receiver [25]. We then divided each dyadic interaction value by the sum of all dyadic values for each interaction type, so that, for each interaction type, the sum of all dyadic indices equalled 1.

### Multiplex network construction

To characterise the multidimensionality of the social relationships of the study individuals, we used *MuxViz* [55] to construct a three-layered undirected weighted multiplex network for each observation period (i.e., one for *period one* and one for *period two*) using the dyadic indices as edge weights. In these two networks, each layer corresponded to a type of interaction. To characterise the temporal dynamics of the social relationships of the study individuals, we used *MuxViz* to construct a two-layered undirected weighted multiplex network for each type of interaction (i.e., one for grooming, one for proximity and one for agonism), in which each layer of the same network corresponded to each observation period. Because we did not observe Tania in *period one*, we constructed the three two-layered multiplex networks using data only from the females observed in both periods (i.e., Camila, Lisala, Limbe, Lolaya, Mirinda and Nefertari), thus re-calculating the dyadic indices excluding Tania and all interactions between her and the other females.

For all networks, in each layer, each node corresponded to one of the focal females and each edge corresponded to the relationship between the individuals represented by the nodes that it connected. Since not all individuals were recorded engaging in all types of interactions, the number of nodes varied across layers. Individual layers are connected to each other through interlayer connections that connect the individuals they have in common. Various network properties are influenced by the attribution of different values to links between layers [56], so we gave a value of 1 to all interlayer connections, making the multiplex networks edge-coloured graphics [25].

For simplicity and clarity, we only present the graphic representation and descriptive results for the two three-layered multiplex networks (i.e., the multiplex networks for *period one* and *period two*). We used *MuxViz* to construct a graphic representation of each network, using edge weight to define edge width and eigenvector versatility to define node size, and to calculate its interlayer edge overlap and individual eigenvector centrality and versatility. Eigenvector centrality measures how well connected and influential to the flow of information each individual is in a single-layer network, based both on the number and strength of her relationships and on the influence of her associates. In a social context, it characterises the social importance/ power of an individual [34, 57]. Eigenvector versatility is the multiplex equivalent to eigenvector centrality and characterises the influence of individuals across different types of interactions [33]. These measures are indicated for small-sized networks, take the weight of the edges into account and are appropriate to investigate the stability of the importance of individuals over time [20, 25, 58].

### Reducibility analysis

We used *MuxViz* to conduct reducibility analysis [59] on the two three-layered multiplex networks to test the prediction that, in a network structure, **(1) the multidimensionality of female mandrill sociality is more accurately represented by considering all three types of interactions separately within the same observation period**, and on the three two-layered networks to test the prediction that, in a network structure, **(2) the multidimensionality of female mandrill sociality is more accurately represented by considering each type of interaction separately for each observation period**.

Reducibility analysis identifies which layers are required to accurately represent the structure of multiplex networks and which ones can be aggregated without loss of information [59]. For each network, *MuxViz* quantified the similarity of all pairs of layers by computing their Jensen-Shannon distance, which varies between 0 (maximum similarity) and 1 (maximum dissimilarity) [59], resulting in a distance matrix. Hierarchical clustering was then performed in the distance matrix with the Ward method, indicating the relatedness of information of all layers. The most similar layers were then aggregated one step at a time, with each aggregation resulting in a reduction of one layer to the multilayer network. In each aggregation, a relative von Neumann entropy function compares how distinguishable the network is from the completely aggregated network. The step where relative entropy is maximal corresponds to the network conformation that preserves the most structural characteristics and, thus, the most non-redundant information [59].

## Results

### Descriptive results

In *period one*, we observed 7 dyads (representing 46.7% of all 15 possible dyads) exchanging a total of 12715 seconds of grooming on an average of 26.49 ± 54.15 seconds of grooming per hour of observation across all possible dyads (mean ± standard deviation). We observed 10 dyads (66.7% of all possible dyads) spending a total of 4038 seconds feeding in proximity of each other on an average of 8.41 ± 13.59 seconds per hour of observation across all possible dyads (mean ± standard deviation). Finally, we observed 15 dyads (100% of all possible dyads) engaging on a total of 419 episodes of agonism (386 supplants and avoidances and 33 aggressive interactions), on an average of 0.87 ± 0.94 episodes per hour of observation across all possible dyads.

In *period two*, we observed 9 dyads (42.9% of all 21 possible dyads) exchanging a total of 9202 seconds of grooming on an average of 20.87 ± 32.36 seconds of grooming per hour of observation across all possible dyads (mean ± standard deviation). We observed 8 dyads (38.1% of all possible dyads) spending a total of 1361 seconds feeding in proximity of each other on an average of 3.09 ± 6.17 seconds per hour of observation across all possible dyads (mean ± standard deviation). Finally, we observed 21 dyads (100% of all possible dyads) engaging on a total of 662 agonistic interactions (619 supplants and avoidances and 43 instances of aggression), on an average of 1.50 ± 1.58 episodes per hour of observation across all possible dyads.

**Fig 1:**
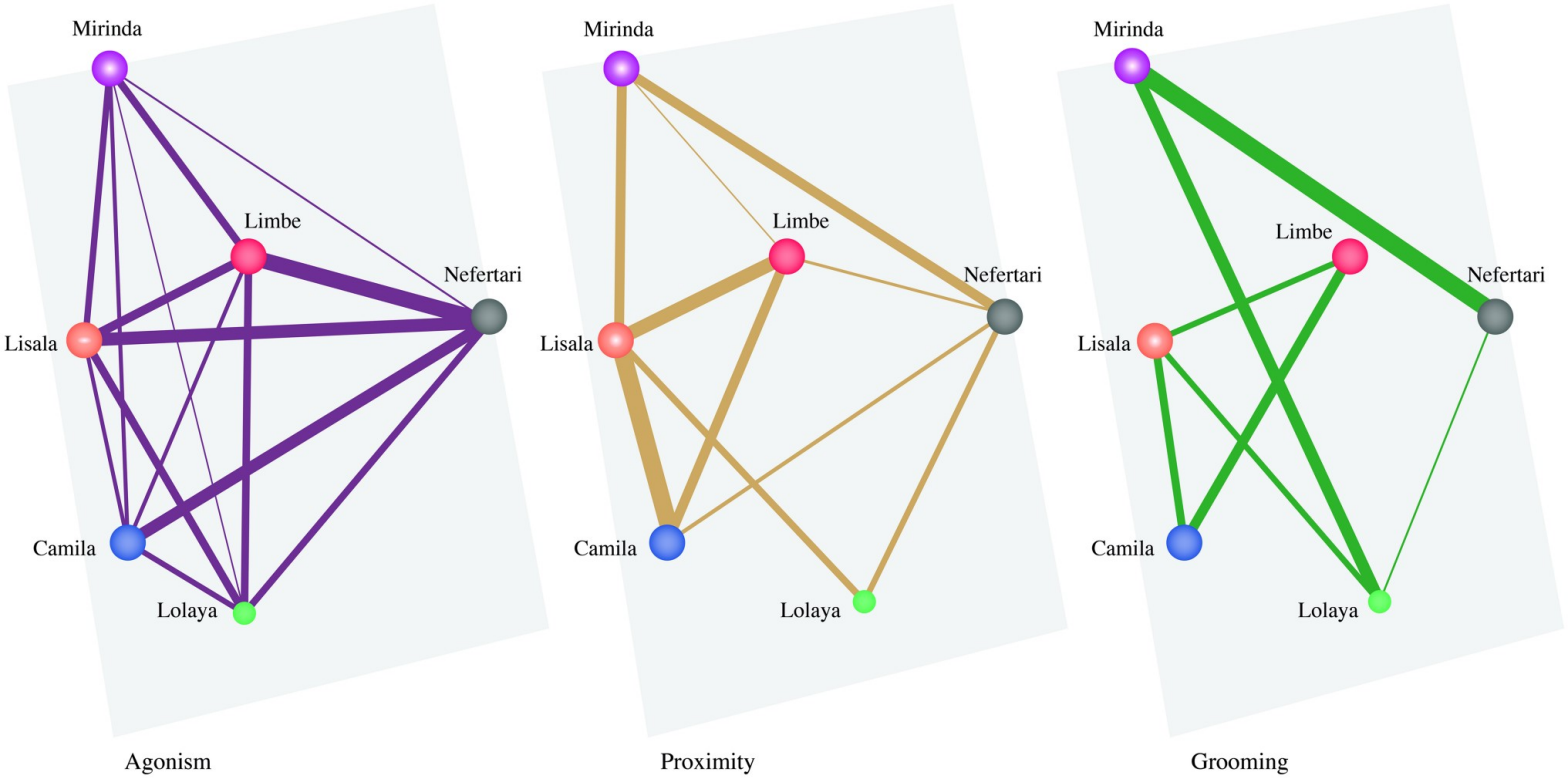
Graphical representation of the multiplex network in *period one*. Tie width is defined by tie strength and node size is defined by eigenvector versatility; to help visualisation, all nodes have different colours, but each node has the same colour across layers, and edges have different colours across layers.

The multiplex network for *period one* was constituted by 6 nodes (i.e., individuals) and 32 intra-layer edges across the three layers and all nodes were found in all three layers (Fig 1). The network had a mean global edge overlap of 3.87% across all layers. The layers that had the highest overlapping value were the grooming and proximity layers (0.31), indicating that individuals who groomed each other were often also found in proximity when feeding. The grooming and agonism layers had the lowest overlapping value (0.08), meaning that individuals that groomed each other tended not to aggress, supplant or avoid each other. The proximity and agonism layers had an overlapping value of 0.20. The most central individuals were Mirinda in the grooming layer, Lisala in the proximity layer and Nefertari in the agonism layer. Nefertari was also the most versatile at the multiplex level.

**Fig 2:**
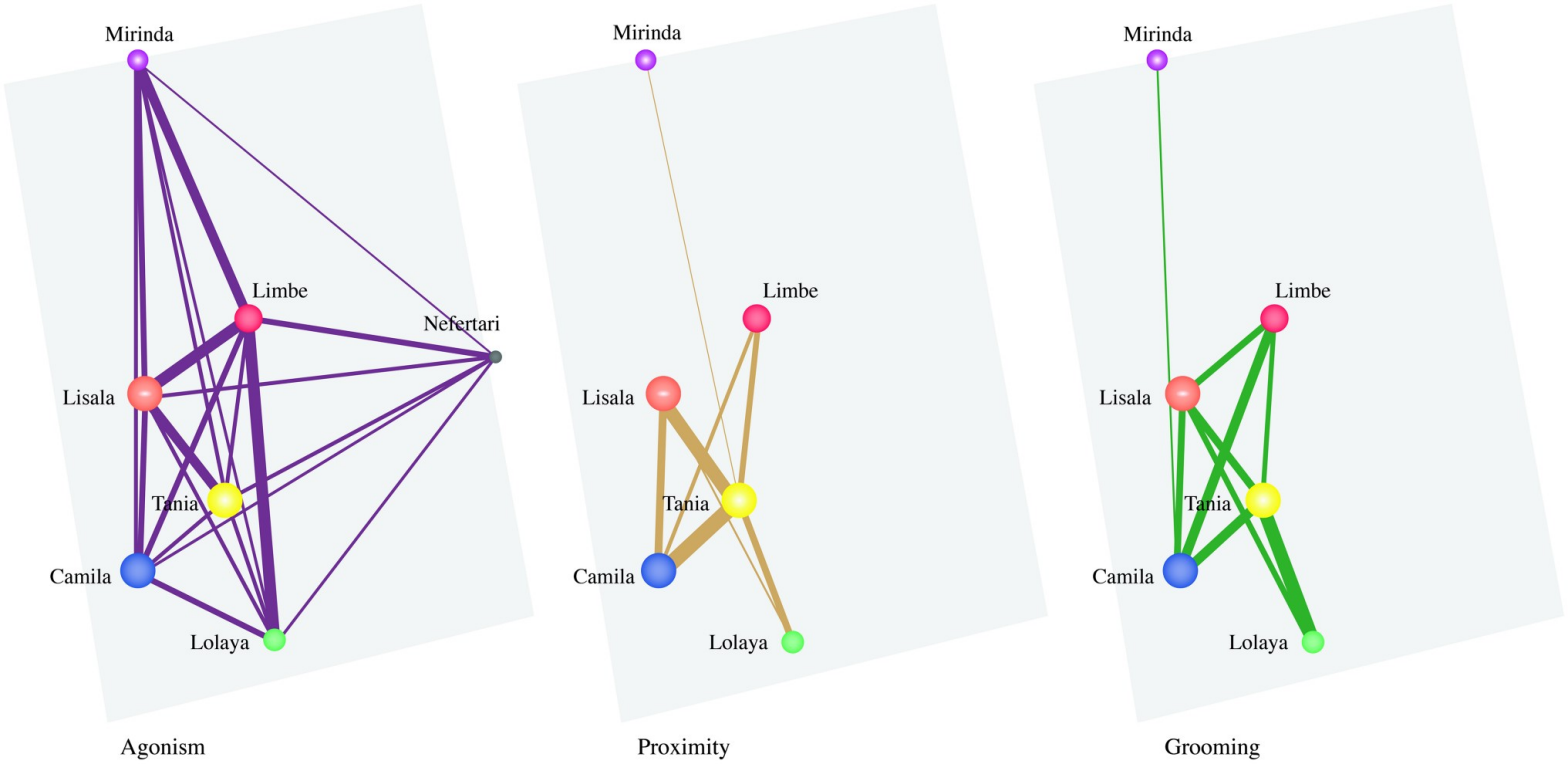
Graphical representation of the multiplex network in *period two*. Tie width is defined by tie strength and node size is defined by eigenvector versatility; to help visualisation, all nodes have different colours, but each node has the same colour across layers, and edges have different colours across layers.

For *period two*, the multiplex network was composed of 7 nodes (i.e., individuals) and a total of 38 intra-layer edges and the agonism layer was the only layer where all nodes were present as Nefertari did not groom or feed in proximity of other females (Fig 2). The network had a mean global edge overlap of 11.68% across the three layers. Similarly to *period one*, the grooming and proximity layers had the highest overlapping value (0.50). The grooming and agonism layers had an overlapping value of 0.37 and the proximity and agonism layers had the lowest overlapping value (0.32). Tania was the most central individual in the grooming and proximity layer and the most versatile at the multiplex level, whereas Limbe was the most central in the agonism layer. All dyadic indices for all interaction types in all networks in *periods one* and *two* (with and without Tania) can be found in S2 and S3 Tables, respectively. All individual eigenvector centrality and versatility measures can be found in S4 Table.

### Reducibility analysis

**Fig 3:**
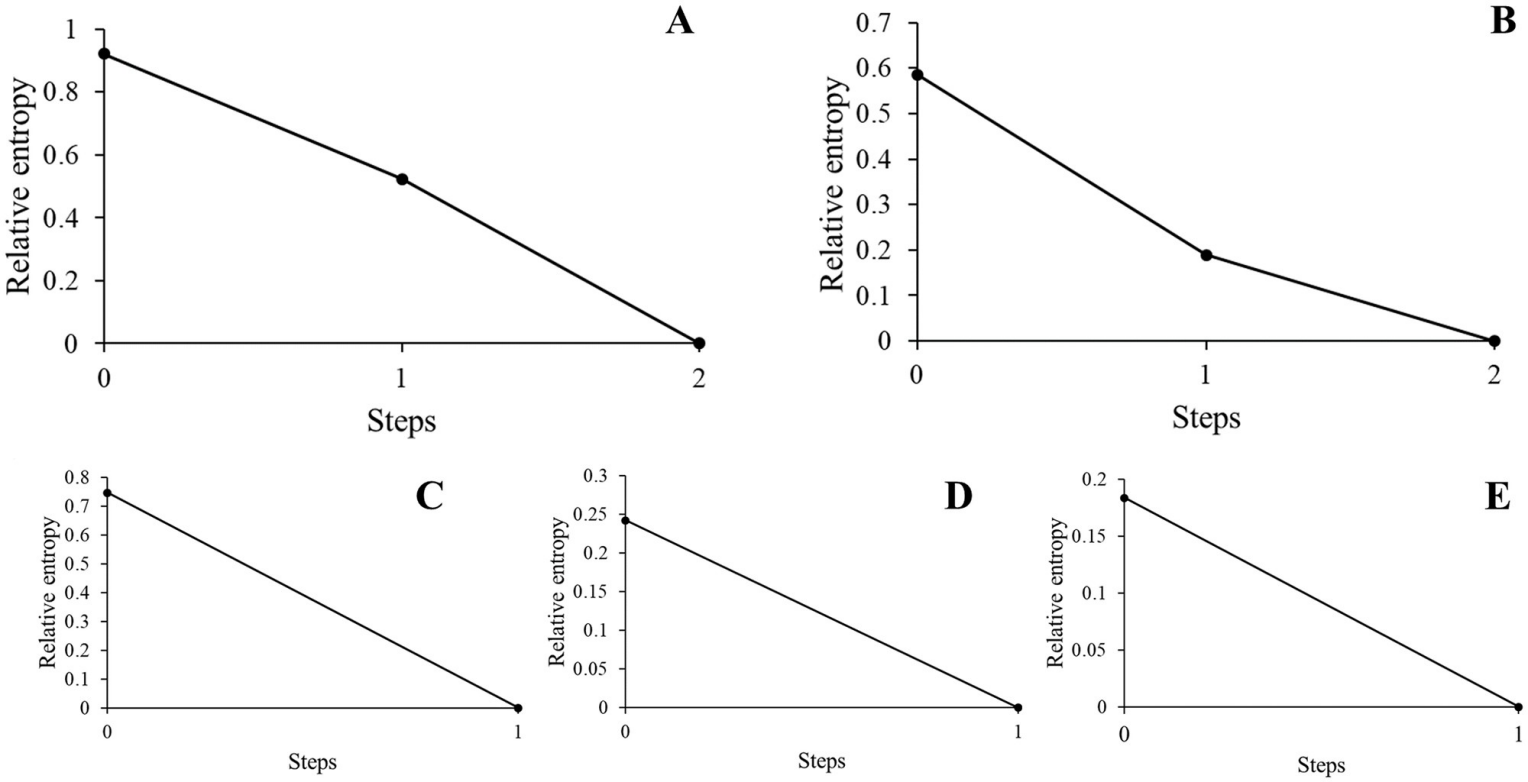
Relative entropy at each aggregation step of the reducibility analysis of all multiplex networks. Each graph represents the relative entropy of the respective multilayer network at each aggregation step compared to the fully aggregated network. All graphs show that the relative entropy is highest at step 0 (i.e., when all layers are considered separately), indicating that all layers contribute non-redundant information to the multilayer structure and, thus, that no reduction should be done. Graph A corresponds to the three-layered network of *period one*, B to the three-layered network of *period two*, C to the two-layered grooming network, D to the two-layered proximity network, and E to the two-layered agonism network.

We found full support for our predictions (1) and (2) as reducibility analyses showed that, for all networks, the relative entropy was highest when all layers were considered separately; the aggregation of any layer would lead to loss of non-redundant information (Fig 3). Jensen-Shannon distance values for each network can be found in S5 Table and relative entropy values at each aggregation step of each reducibility analysis can be found in S6 Table.

## Discussion

Multiplex network analysis characterised the multidimensionality of relationships for these female mandrills within and across two observation periods. Grooming, proximity and agonistic interactions studied at two separate time periods all provided non-redundant information about the social relationships of the study females, supporting our hypothesis. Analysing different social contexts and how these change over time allows for a more comprehensive characterisation of relationships [10], and our results illustrate the potential of multiplex network analyses to incorporate multiple social and temporal dimensions in a network structure relative to standard monolayer network approaches [25].

The results of the reducibility analysis on the multiplex networks of *period one* and *two* support prediction (1) and endorse the use of all interaction types to characterise the social relationships of the study females. Furthermore, in both observation periods, different layers had different edge overlap values, which were globally small, and different individuals were the most central across interaction types and at the multiplex level. It is expected that individuals within primate groups are connected differently and have a varying engagement with different interaction types [23–25]. Thus, each interaction type encompasses potentially variable meaningful information about the relationships between dyads. Primates are socially and cognitively complex animals and it is likely that each type of interaction impacts their social lives uniquely. For example, as observed for Tania in *period two*, a versatile individual may be central in grooming but not in agonism due to the potential fitness consequences of engaging in agonism [23]. The multiplex approach provides an understanding of individual role in structuring relationships that might otherwise not be evident. Overall, our findings suggest that considering various types of interactions separately in a multiplex context allows us to characterise the relationships of female mandrills in a comprehensive way.

Likewise, the reducibility analysis on the grooming, proximity and agonism multiplex networks supported prediction (2), indicating that different layers consisting on the same interaction type over time contain different information, thus endorsing the study of relationships across different time periods. Between periods, the composition of the adult female’s network changed due to the sexual maturation of Tania, which likely contributed to the difference in information between layers. Even though she was excluded from analysis, the way other individuals interact with each other was likely affected by their relationships with Tania. Furthermore, Nefertari’s infant became independent between observation periods, potentially affecting the way other females interacted with her and thus influencing the structure of the networks. Dependent infants are a source of interest to female Old World monkeys and may influence the way other females interact with the infants’ mothers [18]. These findings provide further support to the idea that primate social networks are dynamic structures [15] and that demographic variation influences their structure [17].

SNA-based tools have the potential to identify social isolation and ostracism, such as the case of Nefertari, who died of no apparent cause soon after we finished collecting data. Her absence from the grooming and proximity layers of *period two* suggests that she was already socially excluded. In addition, she received agonism from all other females. Although social exclusion may be adaptive in some circumstances [60], it is often associated with negative fitness consequences [61].

Finally, our approach has limitations. The use of multilayer network analysis in primate studies is still in its infancy and we used undirected data for ease of modelling comparisons across all layers [25]. As such, the direction of grooming and agonism which has biological meaning (e.g., give grooming/ received reduced agonism) was not included. Direction is particularly relevant for agonism, as the fitness consequences of giving and receiving agonism diverge [62]. The biological significance of the direction of grooming and agonistic interactions of the study individuals has been characterised elsewhere [41]; nevertheless, future advances in multilayer network approach need to allow for the integration of directed and undirected interactions as different layers of the same multilayer. Our findings suggest that the differences in the social relationships of the females between periods might be due to demographic variation; however, these may also be due to factors that we were unable to control in our experimental design. Seasonality affects patterns of sociality in Old World monkeys due to reproductive cycling and variation in food availability [14–15], and even though the study females were under contraception and food was equally available in the two periods, it is possible that temperature, day length or other variable conditions due to captive management influenced the relationships of the study females to some extent. Furthermore, the small sample size and observation time per individual reduced the power of the analyses and limit our capacity to interpret and generalise our findings. The captive setting of the study group and our focus on females also limit our capacity to generalise the obtained results to wild groups; however, mandrills are very difficult to follow in the wild [63] and captive studies provide a rare opportunity to investigate their social behaviour.

## Conclusions

Here, we illustrate the exciting potential of multilayer network analysis to integrate various types of interactions and their dynamics in a network structure in order to characterise the social relationships of primates. We found that female mandrills engage in dynamic relationships across interaction types and time, making a valuable contribution to our still limited understanding of mandrill sociality. Our results evidence the dynamic and multidimensional nature of relationships found even in such small captive primate groups.

## Supporting information

S1 Table Description of the types of interaction used to construct the multiplex networks. (DOCX)

S2 Table Indices of the dyadic relationships of the study mandrills for all interaction types in *period one*. (DOCX)

S3 Table Indices of the dyadic relationships of the study mandrills for all interaction types in *period two* with and without Tania. (DOCX)

S4 Table Individual measures of centrality and versatility from multiplex network analysis of periods *one* and *two*. (DOCX)

S5 Table Jensen-Shannon distances for all pairs of layers of all multiplex networks. (DOCX)

S6 Table Relative entropy values at each aggregation step of each reducibility analysis. (DOCX)

S1 Dataset Period one datasets. Datasets include the grooming, proximity, aggression and supplant and avoidance matrices from *period one*.
(DOCX)

S2 Dataset Period two datasets. Datasets include the grooming, proximity, aggression and supplant and avoidance matrices from *period two*.
(DOCX)
