# Supplementary material for: The multidimensionality of female mandrill sociality—A dynamic multiplex network approach
Source: PLoS One. 2020 Apr 13;15(4):e0230942. doi: 10.1371/journal.pone.0230942 (PMC7153875; doi:10.1371/journal.pone.0230942)
Supplement: S1 Table — (DOCX) [file pone.0230942.s001.docx]

| **Interaction type** | **Description** |
| --- | --- |
| Agonism - Aggression | One individual directly attacks another by chasing it, lunging at it, striking it, biting it, or aggressively grabbing it. This interaction is normally accompanied by screams and flight from the receptor. |
| Agonism - Supplant and avoidance | One individual retreats from its location at the sight of another individual approaching. |
| Proximity | One individual is observed within arm’s reach of the focal individual when the focal individual is feeding or foraging (based on [47]). |
| Grooming | One individual uses its own hands or mouth to manipulate the fur of another individual. |
