# Supplementary material for: The multidimensionality of female mandrill sociality—A dynamic multiplex network approach
Source: PLoS One. 2020 Apr 13;15(4):e0230942. doi: 10.1371/journal.pone.0230942 (PMC7153875; doi:10.1371/journal.pone.0230942)
Supplement: S2 Table — (DOCX) [file pone.0230942.s002.docx]

| **Interactants** | | **Agonism** | **Proximity** | **Grooming** |
| --- | --- | --- | --- | --- |
| Camila | Lisala | 0.0167 | 0.3757 | 0.0484 |
| Camila | Limbe | 0.0167 | 0.1384 | 0.1372 |
| Camila | Lolaya | 0.0239 | 0.0000 | 0.0000 |
| Camila | Mirinda | 0.0167 | 0.0000 | 0.0000 |
| Camila | Nefertari | 0.1337 | 0.0129 | 0.0000 |
| Lisala | Limbe | 0.0764 | 0.2494 | 0.0136 |
| Lisala | Lolaya | 0.0525 | 0.0149 | 0.0139 |
| Lisala | Mirinda | 0.0358 | 0.0981 | 0.0000 |
| Lisala | Nefertari | 0.1814 | 0.0000 | 0.0000 |
| Limbe | Lolaya | 0.0668 | 0.0000 | 0.0000 |
| Limbe | Mirinda | 0.0764 | 0.0012 | 0.0000 |
| Limbe | Nefertari | 0.2673 | 0.0072 | 0.0000 |
| Lolaya | Mirinda | 0.0024 | 0.0000 | 0.2804 |
| Lolaya | Nefertari | 0.0286 | 0.0134 | 0.0116 |
| Mirinda | Nefertari | 0.0048 | 0.0889 | 0.4949 |
