# Supplementary material for: The multidimensionality of female mandrill sociality—A dynamic multiplex network approach
Source: PLoS One. 2020 Apr 13;15(4):e0230942. doi: 10.1371/journal.pone.0230942 (PMC7153875; doi:10.1371/journal.pone.0230942)
Supplement: S3 Table — (DOCX) [file pone.0230942.s003.docx]

| **Calculation** | **Interactants** | | **Agonism** | **Proximity** | **Grooming** |
| --- | --- | --- | --- | --- | --- |
| With Tania | Camila | Tania | 0.0076 | 0.3137 | 0.1693 |
|  | Camila | Lisala | 0.0166 | 0.1653 | 0.0950 |
|  | Camila | Limbe | 0.0242 | 0.0301 | 0.1619 |
|  | Camila | Mirinda | 0.0121 | 0.0000 | 0.0053 |
|  | Camila | Lolaya | 0.0393 | 0.0000 | 0.0000 |
|  | Camila | Nefertari | 0.0060 | 0.0000 | 0.0000 |
|  | Tania | Lisala | 0.1208 | 0.3064 | 0.1069 |
|  | Tania | Limbe | 0.0891 | 0.1102 | 0.0217 |
|  | Tania | Mirinda | 0.0196 | 0.0007 | 0.0000 |
|  | Tania | Lolaya | 0.0544 | 0.0713 | 0.2668 |
|  | Tania | Nefertari | 0.0227 | 0.0000 | 0.0000 |
|  | Lisala | Limbe | 0.1692 | 0.0000 | 0.0955 |
|  | Lisala | Mirinda | 0.0347 | 0.0000 | 0.0000 |
|  | Lisala | Lolaya | 0.0378 | 0.0022 | 0.0775 |
|  | Lisala | Nefertari | 0.0136 | 0.0000 | 0.0000 |
|  | Limbe | Mirinda | 0.1571 | 0.0000 | 0.0000 |
|  | Limbe | Lolaya | 0.1148 | 0.0000 | 0.0000 |
|  | Limbe | Nefertari | 0.0453 | 0.0000 | 0.0000 |
|  | Mirinda | Lolaya | 0.0060 | 0.0000 | 0.0000 |
|  | Mirinda | Nefertari | 0.0030 | 0.0000 | 0.0000 |
|  | Lolaya | Nefertari | 0.0060 | 0.0000 | 0.0000 |
| Without Tania | Camila | Lisala | 0.0242 | 0.8364 | 0.2182 |
|  | Camila | Limbe | 0.0352 | 0.1524 | 0.3720 |
|  | Camila | Mirinda | 0.0176 | 0.0000 | 0.0122 |
|  | Camila | Lolaya | 0.0573 | 0.0000 | 0.0000 |
|  | Camila | Nefertari | 0.0088 | 0.0000 | 0.0000 |
|  | Lisala | Limbe | 0.2467 | 0.0000 | 0.2195 |
|  | Lisala | Mirinda | 0.0507 | 0.0000 | 0.0000 |
|  | Lisala | Lolaya | 0.0551 | 0.0112 | 0.1780 |
|  | Lisala | Nefertari | 0.0198 | 0.0000 | 0.0000 |
|  | Limbe | Mirinda | 0.2291 | 0.0000 | 0.0000 |
|  | Limbe | Lolaya | 0.1674 | 0.0000 | 0.0000 |
|  | Limbe | Nefertari | 0.0661 | 0.0000 | 0.0000 |
|  | Mirinda | Lolaya | 0.0088 | 0.0000 | 0.0000 |
|  | Mirinda | Nefertari | 0.0044 | 0.0000 | 0.0000 |
|  | Lolaya | Nefertari | 0.0088 | 0.0000 | 0.0000 |
