# Supplementary material for: The multidimensionality of female mandrill sociality—A dynamic multiplex network approach
Source: PLoS One. 2020 Apr 13;15(4):e0230942. doi: 10.1371/journal.pone.0230942 (PMC7153875; doi:10.1371/journal.pone.0230942)
Supplement: S4 Table — (DOCX) [file pone.0230942.s004.docx]

| **Period** | **Individual** | **Agonism (centrality)** | **Proximity (centrality)** | **Grooming (centrality)** | **Multiplex (versatility)** |
| --- | --- | --- | --- | --- | --- |
| One | Camila | 0.408 | 0.893 | 0.001 | 0.774 |
|  | Limbe | 0.871 | 0.704 | 0.001 | 0.853 |
|  | Lisala | 0.669 | 1.000 | 0.012 | 0.908 |
|  | Lolaya | 0.316 | 0.030 | 0.507 | 0.435 |
|  | Mirinda | 0.246 | 0.197 | 1.000 | 0.862 |
|  | Nefertari | 1.000 | 0.065 | 0.873 | 1.000 |
| Two | Camila | 0.199 | 0.833 | 0.757 | 0.757 |
|  | Limbe | 1.000 | 0.247 | 0.503 | 0.584 |
|  | Lisala | 0.814 | 0.812 | 0.686 | 0.839 |
|  | Lolaya | 0.536 | 0.134 | 0.767 | 0.497 |
|  | Mirinda | 0.566 | 0.001 | 0.010 | 0.135 |
|  | Nefertari | 0.214 | 0.000 | 0.000 | 0.057 |
|  | Tania | 0.649 | 1.000 | 1.000 | 1.000 |
