# Supplementary material for: The multidimensionality of female mandrill sociality—A dynamic multiplex network approach
Source: PLoS One. 2020 Apr 13;15(4):e0230942. doi: 10.1371/journal.pone.0230942 (PMC7153875; doi:10.1371/journal.pone.0230942)
Supplement: S5 Table — (DOCX) [file pone.0230942.s005.docx]

| **Network** | **Layer** | **Proximity** | **Grooming** |
| --- | --- | --- | --- |
| Three-layered network of *period one* | Agonism | 0.401 | 0.487 |
|  | Proximity | - | 0.512 |
| Three-layered network of *period two* | Agonism | 0.46 | 0.376 |
|  | Proximity | - | 0.26 |

| **Network** | **Period** | **One** |
| --- | --- | --- |
| Two-layered agonism network | Two | 0.303 |
| Two-layered proximity network | Two | 0.348 |
| Two-layered grooming network | Two | 0.611 |
|  |  |  |
