# Supplementary material for: The multidimensionality of female mandrill sociality—A dynamic multiplex network approach
Source: PLoS One. 2020 Apr 13;15(4):e0230942. doi: 10.1371/journal.pone.0230942 (PMC7153875; doi:10.1371/journal.pone.0230942)
Supplement: S6 Table — (DOCX) [file pone.0230942.s006.docx]

| **Network** | **Aggregation step** | **Relative entropy** |
| --- | --- | --- |
| Three-layered network of *period one* | 0 | 0.922 |
|  | 1 | 0.523 |
|  | 2 | -4.00^-15^ |
| Three-layered network of *period two* | 0 | 0.586 |
|  | 1 | 0.189 |
|  | 2 | 0 |
| Two-layered agonism network | 0 | 0.184 |
|  | 1 | 0 |
| Two-layered proximity network | 0 | 0.242 |
|  | 1 | 0 |
| Two-layered grooming network | 0 | 0.748 |
|  | 1 | 0 |
|  |  |  |
