# Supplementary material for: The multidimensionality of female mandrill sociality—A dynamic multiplex network approach
Source: PLoS One. 2020 Apr 13;15(4):e0230942. doi: 10.1371/journal.pone.0230942 (PMC7153875; doi:10.1371/journal.pone.0230942)
Supplement: S1 Dataset — Datasets include the grooming, proximity, aggression and supplant and avoidance matrices from period one. (DOCX) [file pone.0230942.s007.docx]

Grooming (in seconds)

|  | **Camila** | **Lisala** | **Limbe** | **Lolaya** | **Mirinda** | **Nefertari** |
| --- | --- | --- | --- | --- | --- | --- |
| **Camila** | 0 | 312 | 894 | 0 | 0 | 0 |
| **Lisala** | 304 | 0 | 65 | 13 | 0 | 0 |
| **Limbe** | 850 | 108 | 0 | 0 | 0 | 0 |
| **Lolaya** | 0 | 164 | 0 | 0 | 2176 | 147 |
| **Mirinda** | 0 | 0 | 0 | 1389 | 0 | 5832 |
| **Nefertari** | 0 | 0 | 0 | 0 | 461 | 0 |

Proximity when feeding (in seconds)

|  | **Camila** | **Lisala** | **Limbe** | **Lolaya** | **Mirinda** | **Nefertari** |
| --- | --- | --- | --- | --- | --- | --- |
| **Camila** | 0 | 1517 | 559 | 0 | 0 | 52 |
| **Lisala** | 1517 | 0 | 1007 | 60 | 396 | 0 |
| **Limbe** | 559 | 1007 | 0 | 0 | 5 | 29 |
| **Lolaya** | 0 | 60 | 0 | 0 | 0 | 54 |
| **Mirinda** | 0 | 396 | 5 | 0 | 0 | 359 |
| **Nefertari** | 52 | 0 | 29 | 54 | 359 | 0 |

Supplants and avoidances (number of episodes)

|  | **Camila** | **Lisala** | **Limbe** | **Lolaya** | **Mirinda** | **Nefertari** |
| --- | --- | --- | --- | --- | --- | --- |
| **Camila** | 0 | 6 | 4 | 4 | 7 | 54 |
| **Lisala** | 0 | 0 | 32 | 20 | 15 | 76 |
| **Limbe** | 0 | 0 | 0 | 15 | 31 | 106 |
| **Lolaya** | 0 | 0 | 1 | 0 | 1 | 12 |
| **Mirinda** | 0 | 0 | 0 | 0 | 0 | 2 |
| **Nefertari** | 0 | 0 | 0 | 0 | 0 | 0 |

Aggression (number of episodes)

|  | **Camila** | **Lisala** | **Limbe** | **Lolaya** | **Mirinda** | **Nefertari** |
| --- | --- | --- | --- | --- | --- | --- |
| **Camila** | 0 | 1 | 3 | 6 | 0 | 2 |
| **Lisala** | 0 | 0 | 0 | 2 | 0 | 0 |
| **Limbe** | 0 | 0 | 0 | 12 | 1 | 6 |
| **Lolaya** | 0 | 0 | 0 | 0 | 0 | 0 |
| **Mirinda** | 0 | 0 | 0 | 0 | 0 | 0 |
| **Nefertari** | 0 | 0 | 0 | 0 | 0 | 0 |
