# Supplementary material for: The multidimensionality of female mandrill sociality—A dynamic multiplex network approach
Source: PLoS One. 2020 Apr 13;15(4):e0230942. doi: 10.1371/journal.pone.0230942 (PMC7153875; doi:10.1371/journal.pone.0230942)
Supplement: S2 Dataset — Datasets include the grooming, proximity, aggression and supplant and avoidance matrices from period two. (DOCX) [file pone.0230942.s008.docx]

Grooming (in seconds)

|  | **Camila** | **Tania** | **Lisala** | **Limbe** | **Mirinda** | **Lolaya** | **Nefertari** |
| --- | --- | --- | --- | --- | --- | --- | --- |
| **Camila** | 0 | 1293 | 243 | 79 | 0 | 0 | 0 |
| **Tania** | 265 | 0 | 36 | 0 | 0 | 121 | 0 |
| **Lisala** | 631 | 948 | 0 | 0 | 0 | 134 | 0 |
| **Limbe** | 1411 | 200 | 879 | 0 | 0 | 0 | 0 |
| **Mirinda** | 49 | 0 | 0 | 0 | 0 | 0 | 0 |
| **Lolaya** | 0 | 2334 | 579 | 0 | 0 | 0 | 0 |
| **Nefertari** | 0 | 0 | 0 | 0 | 0 | 0 | 0 |

Proximity when feeding (in seconds)

|  | **Camila** | **Tania** | **Lisala** | **Limbe** | **Mirinda** | **Lolaya** | **Nefertari** |
| --- | --- | --- | --- | --- | --- | --- | --- |
| **Camila** | 0 | 427 | 225 | 41 | 0 | 0 | 0 |
| **Tania** | 427 | 0 | 417 | 150 | 1 | 97 | 0 |
| **Lisala** | 225 | 417 | 0 | 0 | 0 | 3 | 0 |
| **Limbe** | 41 | 150 | 0 | 0 | 0 | 0 | 0 |
| **Mirinda** | 0 | 1 | 0 | 0 | 0 | 0 | 0 |
| **Lolaya** | 0 | 97 | 3 | 0 | 0 | 0 | 0 |
| **Nefertari** | 0 | 0 | 0 | 0 | 0 | 0 | 0 |

Supplants and avoidances (number of episodes)

|  | **Camila** | **Tania** | **Lisala** | **Limbe** | **Mirinda** | **Lolaya** | **Nefertari** |
| --- | --- | --- | --- | --- | --- | --- | --- |
| **Camila** | 0 | 4 | 9 | 15 | 8 | 19 | 4 |
| **Tania** | 0 | 0 | 79 | 55 | 13 | 33 | 10 |
| **Lisala** | 0 | 1 | 0 | 109 | 23 | 23 | 7 |
| **Limbe** | 0 | 0 | 0 | 0 | 103 | 67 | 27 |
| **Mirinda** | 0 | 0 | 0 | 0 | 0 | 4 | 2 |
| **Lolaya** | 0 | 0 | 0 | 0 | 0 | 0 | 4 |
| **Nefertari** | 0 | 0 | 0 | 0 | 0 | 0 | 0 |

Aggression (number of episodes)

|  | **Camila** | **Tania** | **Lisala** | **Limbe** | **Mirinda** | **Lolaya** | **Nefertari** |
| --- | --- | --- | --- | --- | --- | --- | --- |
| **Camila** | 0 | 1 | 2 | 1 | 0 | 7 | 0 |
| **Tania** | 0 | 0 | 0 | 3 | 0 | 3 | 5 |
| **Lisala** | 0 | 0 | 0 | 3 | 0 | 2 | 2 |
| **Limbe** | 0 | 1 | 0 | 0 | 1 | 9 | 3 |
| **Mirinda** | 0 | 0 | 0 | 0 | 0 | 0 | 0 |
| **Lolaya** | 0 | 0 | 0 | 0 | 0 | 0 | 0 |
| **Nefertari** | 0 | 0 | 0 | 0 | 0 | 0 | 0 |
